# Celecoxib Inhibits the Lytic Activation of Kaposi’s Sarcoma-Associated Herpesvirus through Down-Regulation of RTA Expression by Inhibiting the Activation of p38 MAPK

**DOI:** 10.3390/v7052268

**Published:** 2015-05-05

**Authors:** Jungang Chen, Liangyu Jiang, Ke Lan, Xulin Chen

**Affiliations:** 1 State Key Laboratory of Virology, Wuhan Institute of Virology, Chinese Academy of Sciences, Wuhan 43001, Hubei, China; E-Mails: cjgkaoyan@163.com (J.C.); jiangliangyu1988@126.com (L.J.); 2 University of Chinese Academy of Sciences, Beijing 100049, China; 3 Key Laboratory of Molecular Virology and Immunology, Institute Pasteur of Shanghai, Shanghai Institutes for Biological Sciences, Chinese Academy of Sciences, Shanghai 200031, China; E-Mail: lanke@sibs.ac.cn

**Keywords:** Kaposi’s sarcoma associated herpesvirus, celecoxib, RTA, p38 MAPK

## Abstract

Kaposi’s sarcoma associated herpesvirus (KSHV) is the etiologic agent of Kaposi’s sarcoma (KS), primary effusion lymphoma (PEL), and multicentric Castleman’s disease (MCD). KSHV’s lytic replication cycle is critical for the pathogenesis of KSHV-associated diseases. Despite recent progress in the development of treatments for KSHV associated malignancies, these therapies are not completely efficacious and cause side effects. Therefore, more effective therapies with antiviral agents against KSHV are urgently needed. In this study, we identified celecoxib as an antiviral agent against KSHV. Our data suggest that celecoxib inhibits the lytic activation of KSHV through the down-regulation of the expression of the lytic switch protein, replication and transcription activator (RTA), by inhibiting the activation of p38 MAPK. Therefore, celecoxib may provide a candidate inhibitor for the therapeutic research of KSHV-related malignancies.

## 1. Introduction

Kaposi’s sarcoma-associated herpesvirus (KSHV), also referred to as human herpesvirus 8, was isolated from the Kaposi’s sarcoma lesions of patients with AIDS and is a double-strand DNA virus classified as a member of the gamma 2-herpesvirus subfamily [1,2]. Previous studies confirmed KSHV as the etiologic agent of Kaposi’s sarcoma (KS), primary effusion lymphoma (PEL), and multicentric Castleman’s disease (MCD) [1,3]. Kaposi’s sarcoma presents with cutaneous lesions that are found in immunosuppressed patients and most frequently in patients infected with HIV. However, despite recent progress in the development of therapeutics for KSHV associated malignancies, ranging from chemotherapy and radiotherapy to highly active antiretroviral therapy, these therapies are not completely efficacious [4]. Therefore, more effective therapies with direct-acting antiviral agents against KSHV remain urgently needed.

KSHV has a genome of approximately 170 kb and encodes nearly 90 open reading frames (ORFs). Similar to other herpesviruses, KSHV has two alternative life cycle programs for the infection of host cells, the latent and lytic phases [5,6]. Generally, latency infection is established and persists in the host following KSHV primary infection, with only a small population of cells undergoing spontaneous lytic replication [7,8]. During the latent infection, only a limited number of latent associated genes, such as *LANA*, *K13*, *vCyclin*, and *kaposin*, are expressed to facilitate viral latent replication and maintain KSHV genome stabilization by regulating the host microenvironment and inhibiting lytic gene expression [9]. LANA is a pivotal viral factor for regulating maintenance and segregation of the viral genome during latency, and it is also essential for cell survival [10,11]. In the lytic phase, the majority of viral genes are highly expressed to facilitate genomic DNA replication and mature virion production. Following activation from the latency to the lytic cycle, most viral genes are expressed in an orderly fashion based on the time of expression, defined as immediate-early (IE), early and late genes [6,12,13]. An immediate-early gene, RTA, is the molecular switch for KSHV activation and is sufficient to commence lytic replication and initiate the lytic protein cascade to produce abundant mature progeny [12,14]. Therefore, identifying the mechanisms of activation of the RTA promoter is beneficial for understanding KSHV latency and activation.

The activity of the RTA promoter is determined by a number of transcriptional factors and specific cellular signaling pathways, involving protein kinase C (PKC), AP-1, MAPK, NF-κB, the cellular cyclic AMP/protein kinase A (PKA) [15,16,17,18,19]. MAPK pathways, including p38, ERK and JNK pathways play a crucial role in KSHV activation. In the primary infection of primary human umbilical vascular endothelial cells (HUVEC), KSHV activates ERK, JNK and p38 pathways, all of which activate AP-1 to modulate KSHV infection and lytic replication [20,21]. Similarly, all three MAPK pathways are required for 12-O-tetradecanoyl-phorbol-13-acetate (TPA)-induced KSHV reactivation in latent KSHV-infected cells by activating AP-1 to regulate RTA promoter activity and lytic replication. Furthermore, treatment with ERK, JNK and p38 inhibitors suppresses TPA-induced KSHV lytic replication and RTA promoter activity [22,23,24].

Increasing evidence suggests that the lytic replication of KSHV may be essential for the development and maintenance of KSHV-associated neoplasms [25]. The screening and identification of new drugs that target KSHV lytic replication is critical for the prevention and treatment of KSHV-induced malignancies. In this work, we determined that celecoxib has antiviral activity against the lytic replication of KSHV. Further study revealed that celecoxib inhibits KSHV lytic replication via a COX-2-independent mechanism. Celecoxib inhibits the phosphorylation of p38 MAPK, resulting in a decrease of RTA expression. Our data suggest that celecoxib may be beneficial for the clinical research of KSHV-associated diseases.

## 2. Materials and Methods

### 2.1. Cell Culture and Chemical Agents

BCBL-1 cells were grown in RPMI 1640 medium supplemented with 10% fetal bovine serum (FBS, Gibco-BRL, Gaithersburg, MD, USA). For the lytic replication of KSHV, BCBL-1 cells were induced with 20 ng/mL 12-O-tetradecanoyl-phorbol-13-acetate (PMA/TPA) (Beyotime Institute of Biotechnology, Wuhan, China). Human iSLK.219 cells, derived from iSLK cells, were latently infected with a recombinant rKSHV.219 virus and also contained a doxycycline-inducible RTA [26]. The rKSHV.219 expresses the red fluorescent protein (RFP) from the KSHV lytic PAN promoter, the green fluorescent protein (GFP) from the EF-1α promoter [27]. Human iSLK.219 cells were cultured in DMEM supplemented with 10% FBS. The cells were reactivated with 1 µg/mL doxycycline (Dox) and 1.2 mM sodium butyrate (NaB). The chemicals used for cell culture were purchased from Invivogen (San Diego, CA, USA). Celecoxib was purchased from Sigma-Aldrich (St. Louis, MO, USA), NS-398, SP600125 and SB203580 were purchased from Beyotime Institute of Biotechnology, and nimesulide and cidofovir (CDV) were obtained from Selleck Chemicals (Shanghai, China). All drugs were dissolved in dimethylsulfoxide (DMSO).

### 2.2. Cytotoxicity Assay

BCBL-1 cells were induced with TPA for 3 h, washed with phosphate-buffered saline (PBS) and seeded in 96-well plates (2.5 × 10^5^/mL). Then cells were exposed to various concentrations of the indicated compounds or equivalent solvent and the cell viability was assessed at 72 h post TPA-induction by CellTiter-Glo^®^ Luminescent Cell Viability Assay (Promega, Fitchburg, WI, USA) according to the manufacturer’s protocol. The luminescent signal was measured using the Envison 2102 Multilabel Reader (Perkin Elmer, Waltham, MA, USA). For iSLK.219 cells, cells grown to 70% confluence 96-well tissue culture plates were treated with indicated concentrations of compounds in present of Dox and NaB. Then, cell viability was assessed at 48 h as described above. The 50% cytotoxic concentration (CC_50_) for each compound was calculated from these dose-response curves using Graphpad5.0 Prism. The results were presented as mean values with standard deviations (*n =* 3).

### 2.3. Infectivity Assay

The supernatants from iSLK.219-treated or untreated with the compounds in the presence of Dox and NaB were collected at 48 h. Then, the supernatants were used to infect the 293T cells seeded in a 96-well plate at 70% confluency by spinoculation as previously reported using centrifugation at 1500 × *g* for 60 min [28]. The supernatants were then removed and replaced with flash DMEM medium. At 48 h, the expression of GFP per well in 293T cells was detected and analyzed using the Operetta High-Content Screening System (HCS) (Perkin Elmer). Nine image fields per well were recorded by the automated microscope based HCS and the GFP intensity per well was calculated using the Harmony 3.5 software (Perkin Elmer). Data were normalized as the fold change compared to the DMSO control. The results are presented as the mean values with standard deviations (*n =* 3).

### 2.4. Quantitative PCR (qPCR) and Quantitative Reverse Transcription-PCR (RT-qPCR)

KSHV genomic DNA was isolated as previously described [29]. KSHV virion-associated DNA was isolated from KSHV particles as previously described [30]. All qPCR assays were performed using a Bio-Rad CFX96 Touch™ Real-Time PCR detection system using the iTaq™ Universal SYBR^®^ Green Supermix (Bio-Rad) with primers directed to the ORF73 gene (forward, 5'-CCGAGGACGAAATGGAAGTG-3' and reverse, 5'-GGTGATGTTCTGAGTACATAGCGG-3') [31]. The intracellular viral genomic DNA in each sample was normalized to the amount of the GAPDH gene also determined by qPCR by using primers (forward, 5'-GCTCCCTCTTTCTTTGCAGCAAT-3' and reverse, 5'-TACCATGAGTCCTTCCACGATAC-3') [32].

KSHV virion-associated DNA in the supernatants was measured with primers directed to ORF73 as described above. The production ratio of the KSHV virion upon treatment with the compounds was normalized to the production of the TPA un-induced samples.

Transcripts of genes of interest were also measured by RT-qPCR. The sequences and usage parameters for the primers for the quantification of tested genes were previously described [33]. The data was normalized to the actin housekeeping gene expression using primers directed to actin gene (forward, 5'-ATCGTGCGTGACATTAAGGAG-3' and reverse, 5'-GGAAGGAAGGCTGGAAGAGT-3') [34].

### 2.5. Western Blot Analysis

The expression or activity of the proteins of interest were detected by WB using protein-specific antibody as described previously [9]. The rabbit anti-phosphorylation p38 MAPK (Thr180/Tyr182), rabbit anti-phosphorylation p44/42 MAPK (Erk1/2) (Thr202/Tyr204) monoclonal antibody and rabbit anti-phosphorylation STAT3 (Phospho-Tyr705) antibody were provided by Cell signaling technologies (Danvers, MA, USA). Mouse anti-p38, rabbit anti-ERK, anti-STAT3 and mouse anti-GADPH and anti-β-actin antibodies were purchased from Beyotime Institute of biotechnology. Rabbit anti-LANA [9] and anti-RTA [35] antibodies were prepared by our laboratory.

### 2.6. Fluorescence Detection and Analysis

To assess the effect of the indicated compounds on KSHV reactivation, iSLK.219 cells were analyzed for the fluorescence intensity of RTA-driven RFP using the Operetta High-Content Screening System (HCS) (Perkin Elmer). The cells were seeded on black walled and clear bottomed 96-well plates (Coring Incorporated, Corning, NY, USA) and were treated with or without compounds as described above in complete DMEM supplemented with DOX and NaB for 24 h, followed by the detection of RFP and GFP expression using HCS and quantitative analysis with the Harmony3.5 software (Perkin Elmer). Nine image fields per well were recorded and used for the quantitative analysis of the intensity of RFP and GFP following the software protocol. Data were normalized as the fold change compared to the DMSO control. The results are presented as the mean values with standard deviations (*n =* 3).

### 2.7. Luciferase Reporter Assay

The 1083 bp KSHV RTA promoter was inserted in the pGL3-basic vector (Promega) between the *Sac*I and *Nhe*I sites to create the pGL3-RTA luciferase reporter construct using specific primers (forward, 5'-GAAGAGCTCCTTCCACGTTGATCCGGCTT-3' and reverse, 5'-GATAGCTAGCTTGTGGCTGCCTGGACAGT-3'). The vector pCR3.1-ORF50 encoding the full-length of RTA gene was a kind gift from Yan Yuan (University of Pennsylvania School of Dental Medicine, Philadelphia, PA, USA). The pRL-TK vector was purchased from Promega.

HEK 293 cells seeded in 24-well plates were either treated with the test compounds or co-transfected with pGL3-RTA, pCR3.1-ORF50 and pRL-TK as a transfection efficiency control using Lipofectamine 2000 (Invitrogen, Waltham, MA, USA). The cells were then cultured in the presence of various concentrations of the test compounds. After different incubation times, the luciferase activity was determined in the cell lysates using a dual luciferase assay system (Promega) as recommended by the manufacturer. The levels of luciferase are expressed relative to the levels in the cells without test compound induction. The results are presented as the mean values with standard deviations (*n =* 3).

## 3. Results

### 3.1. Effect of Celecoxib on the Lytic and Latent Replication of KSHV in BCBL-1 Cells

**Figure 1:**
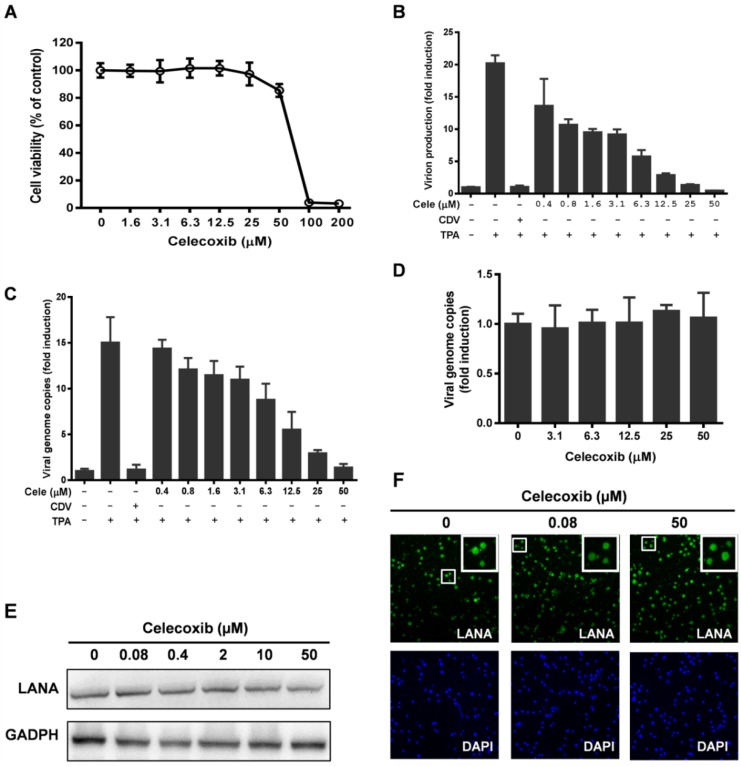
The effects of celecoxib on lytic and latent replication of Kaposi’s sarcoma-associated herpesvirus (KSHV) in BCBL-1 cells. (**A**–**C**) The effects of celecoxib on lytic replication of KSHV. BCBL-1 cells were lytically induced by TPA for 3h and then exposed to the indicated concentrations of celecoxib (cele) for 72 h; Then, the cell viability was determined by CellTiter-Glo^®^ Luminescent Cell Viability Assay (**A**); The virion production was determined by quantitative PCR (qPCR) analysis of the viral DNA extracted from equivalent supernatants using primers specific for the KSHV ORF73 gene (**B**); The copy numbers of the intracellular viral DNA were directly extracted from cells and were quantified by qPCR using primers specific for the KSHV ORF73 gene. Then each sample was normalized to the amount of the GAPDH gene (**C**); CDV (20 µM) was used as a positive control; (**D**–**F**) The effects of celecoxib on latent replication of KSHV. BCBL-1 cells were treated with celecoxib in the absence of TPA for 72 h. Then, the intracellular viral DNA copies was measured by qPCR (**D**), LANA protein expression were detect by Western blotting (**E**) and indirect immunofluorescence assay (**F**) using anti-LANA as described in Methods; Data in (**B**) and (**C**) were normalized as the fold change compared with the TPA non-induced control; Data in (**D**) were normalized as the fold change compared with DMSO treatment control. The results are presented as the mean values with standard deviations (*n =* 3).

To confirm the antiviral activity of celecoxib, BCBL-1 cells induced by TPA for 3 h were exposed to increasing concentrations of celecoxib. Cytotoxicity and inhibitory function analyses were performed at 72 h post-incubation. First, the 50% cytotoxic concentration (CC_50_) for TPA-induced BCBL-1 cells was determined as 58.03 µM, and the maximum non-toxic concentration was 25 µM (Figure 1A). Then, KSHV virion production and viral DNA replication were examined in parallel to the cytotoxicity assay on BCBL-1 cells using qPCR analysis. As shown in Figure 1B, celecoxib effectively reduced the production of progeny virion with a 50% inhibitory concentration (IC_50_) of 2.34 µM. The selectivity index (SI = CC_50_/IC_50_) of celecoxib for KSHV virion production was 24.8. The numbers of viral genome copies in the cells were dose-dependently decreased compared to the control (Figure 1C). At the maximum non-toxic concentration (25 µM), celecoxib reduced lytic replication and virion production by 80.5% and 93.3%, respectively. As expected, CDV completely inhibited the lytic replication and virion production of KSHV (Figure 1B,C) as described previously [36].

The maintenance of KSHV episomes in latency is closely related to its lytic replication program. To determine whether the inhibitory effect of celecoxib on KSHV lytic replication is due to the loss of latent KSHV episomes, BCBL-1 cells were exposed to celecoxib in the absent of TPA induction, and 72 h post-treatment, the KSHV copy numbers in cells was determined by qPCR. The results showed that at concentrations ranging from 5 to 50 µM, celecoxib minimally affected the maintenance of KSHV episomes in latency (Figure 1D). Next, we assessed the expression of LANA, a pivotal regulator of genome maintenance and cell survival in latency [10,37]. Western blotting and indirect immunofluorescence assay revealed that celecoxib treatment had no appreciable effect on this protein (Figure 1E,F), further indicating that celecoxib has a disproportionate impact on KSHV lytic replication compared to KSHV latency under our experimental conditions.

### 3.2. The Antiviral Activity of Celecoxib is Independent of the COX-2 Pathway

**Figure 2:**
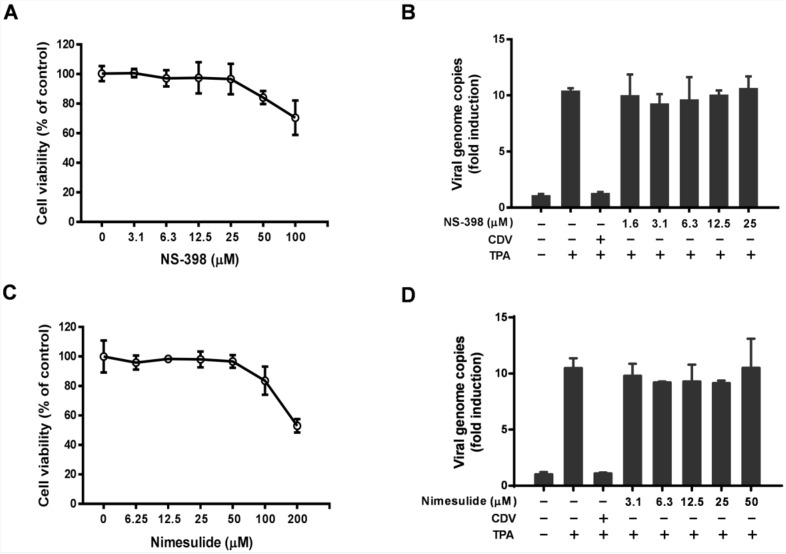
COX-2 inhibitors, NS-398 and nimesulide, have no effect on KSHV lytic replication in BCBL-1 cells. The cells induced with TPA for 3 h were treated with the test compounds for 72 h. Then, the effect of NS-398 and nimesulide on cell viability (**A**,**C**) and the number of DNA copies (**B**,**D**) were measured as described in the Methods. CDV (20 µM) is a positive control for inhibiting viral DNA replication. Data were normalized as the fold change compared with the no-TPA induced control. The results are presented as the mean values with standard deviations (*n =* 3).

Because celecoxib is a COX-2 inhibitor, we determined whether the COX-2 pathway is involved in KSHV lytic replication. Two other selective COX-2 inhibitors, NS-398 and nimesulide, were tested for possible inhibition of the lytic replication of KSHV. First, the cytotoxicity of NS-398 and nimesulide on TPA-induced BCBL-1 cells was examined as described in the Methods section (Figure 2A,C). The copy number of viral DNA in the TPA-induced BCBL-1 cells treated with non-toxic concentrations of the indicated COX-2 inhibitor as determined by qPCR remained unchanged, indicating the NS-398 (Figure 2B) and nimesulide (Figure 2D) exerted a minimal effect against KSHV lytic replication. Despite this, the KSHV polymerase inhibitor, CDV, completely inhibited lytic DNA synthesis. Taken together, our results indicate that the antiviral activity of celecoxib is independent of COX-2 inhibition, and may be an off-target effect via a COX-2-independent mechanism.

### 3.3. Celecoxib Affects the Early Stages of KSHV Reactivation from Latency in iSLK.219 Cells

**Figure 3:**
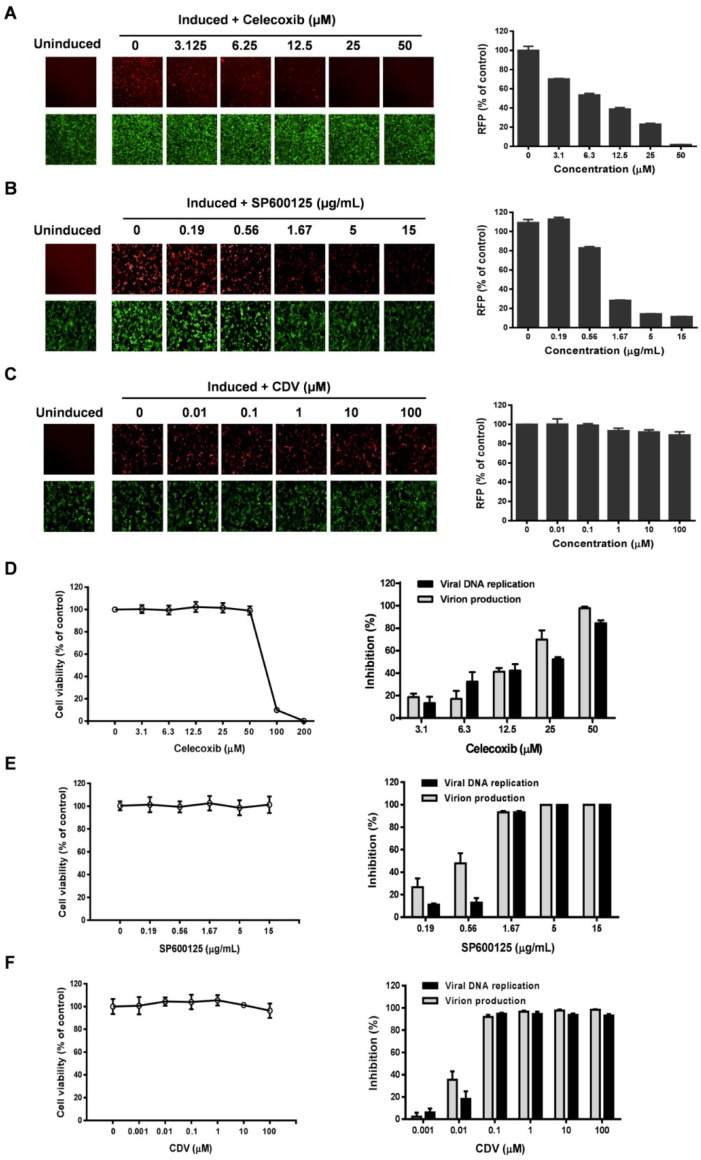
Celecoxib affects the early stages of KSHV reactivation from latency in iSLK.219 cells. The iSLK.219 cells were lytically induced with Dox (1 µg/mL) and NaB (1.2 mM) in the presence of various concentrations of test compounds for 24 h. The effect of celecoxib (**A**), SP600125 (**B**) and CDV (**C**) on the expression and of RFP or GFP were detected by fluorescence microscopy (left panel), and the fluorescence intensity of RFP was quantified with the HCS system as described in the Methods (right panel); The cytotoxicity and the inhibition of viral DNA replication and production of mature virions of celecoxib (**D**), SP600125 (**E**) and CDV (**F**) to iSLK.219 cells were determined by CellTiter-Glo^®^ Luminescent Cell Viability Assay, qPCR (black bars) and the infectivity assay (grey bars) as described in the Methods. Images from nine fields per well were digitized to determine the total intensity using the Harnomy3.5 software. Data were normalized as the relative fluorescence intensity compared to the DMSO control and percent of virion inhibition were calculated relative to the DMSO control. The results are presented as the mean values with standard deviations (*n =* 3).

To identify the stages targeted by celecoxib during viral replication, iSLK.219 cells were used to determine the effect of celecoxib on the stages of KSHV reactivation, DNA replication and virion production. iSLK.219 cells harbor the rKSHV.219 virus that encodes green fluorescent protein (GFP) under control of the elongation factor 1α promoter (EF-1α) and contain a PAN promoter-driven RFP reporter [26,27]. The PAN promoter-driven RFP reporter directly stimulated by RTA is used as a maker of early gene expression and reactivation [27,38]. Therefore, iSLK.219 cells were treated with celecoxib or control compounds in the presence of Dox and NaB. Then, the expression of RFP, the number of viral DNA copies and the production of infectious virions were determined as described in the Methods section. The inhibition profile of celecoxib was compared to reference compounds with a known mechanism. As shown in Figure 3A, celecoxib dose-dependently reduced RFP expression as determined by fluorescence assay (left panel) and the intensity analysis of RFP (right panel). Similarly, SP600125, an inhibitor targeting the early stages of KSHV reactivation, also significantly inhibits RFP expression (Figure 3B); however, CDV, a KSHV polymerase inhibitor, has a minimal inhibitory effect on RFP expression (Figure 3C). In comparison, celecoxib displays an inhibition profile similar to SP600125, indicating that celecoxib may target the early stages of KSHV reactivation. The blockage of reactivation affects DNA replication and virion production. As expected, SP600125 and CDV inhibited viral DNA replication and virion production at non-toxic concentrations (Figure 3E,F). As shown in Figure 3D, the number of viral DNA copies and the production of infectious virions were also reduced by celecoxib at non-toxic concentrations, which is consistent with its inhibitory effect on reactivation. To better define the targeted stage (s) of celecoxib, we added celecoxib and two reference compounds CDV and SP600125 at different times post-induction in iSLK.219 cells to detect the reactivation and the virion production. The results showed that the later celecoxib were added, the less inhibitory effects on RFP expression and virion production were observed (Figure S1A,B). In comparison, SP600125 (Figure S1E,F) but not CDV (Figure S1C,D) displayed similar inhibitory profile, further suggesting the targeted stage (s) of celecoxib are early phase of KSHV reactivation.

**Figure 4:**
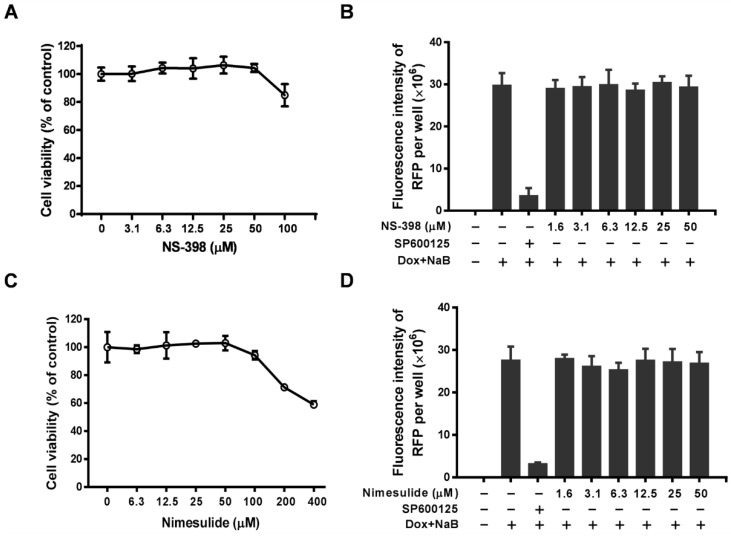
COX-2 inhibitors, NS-398 and Nimesulide, have no effect on KSHV reactivation in iSLK.219 cells. The cells were lytically induced with Dox and NaB in the presence of various concentrations of test compounds and incubated for 24 h. The effect of NS-398 and nimesulide on cytotoxicity to cells (**A**,**C**) and the fluorescence intensity of RFP (**B**,**D**) were detected and digitalized as described in the Methods. SP600125 (5 µg/mL) is a positive control for inhibiting KSHV reactivation. Data were normalized as the relative fluorescence intensity compared to the DMSO control. The results are presented as the mean values with standard deviations (*n =* 3).

To confirm whether KSHV reactivation is associated with the COX-2 pathway, the COX-2 inhibitors, NS-398 and nimesulide, were used to determine their effects on the reactivation of KSHV in iSLK.219 cells. As shown in Figure 4, and as expected, SP600125 completely inhibited the RFP expression as described previously, however NS-398 and nimesulide had no inhibitory effects on RFP expression even at the maximum non-toxic concentrations, suggesting that the inhibitory effect of celecoxib on KSHV reactivation is specific to the compound and is independent of the COX-2 pathway.

### 3.4. Celecoxib Suppresses RTA Expression

**Figure 5:**
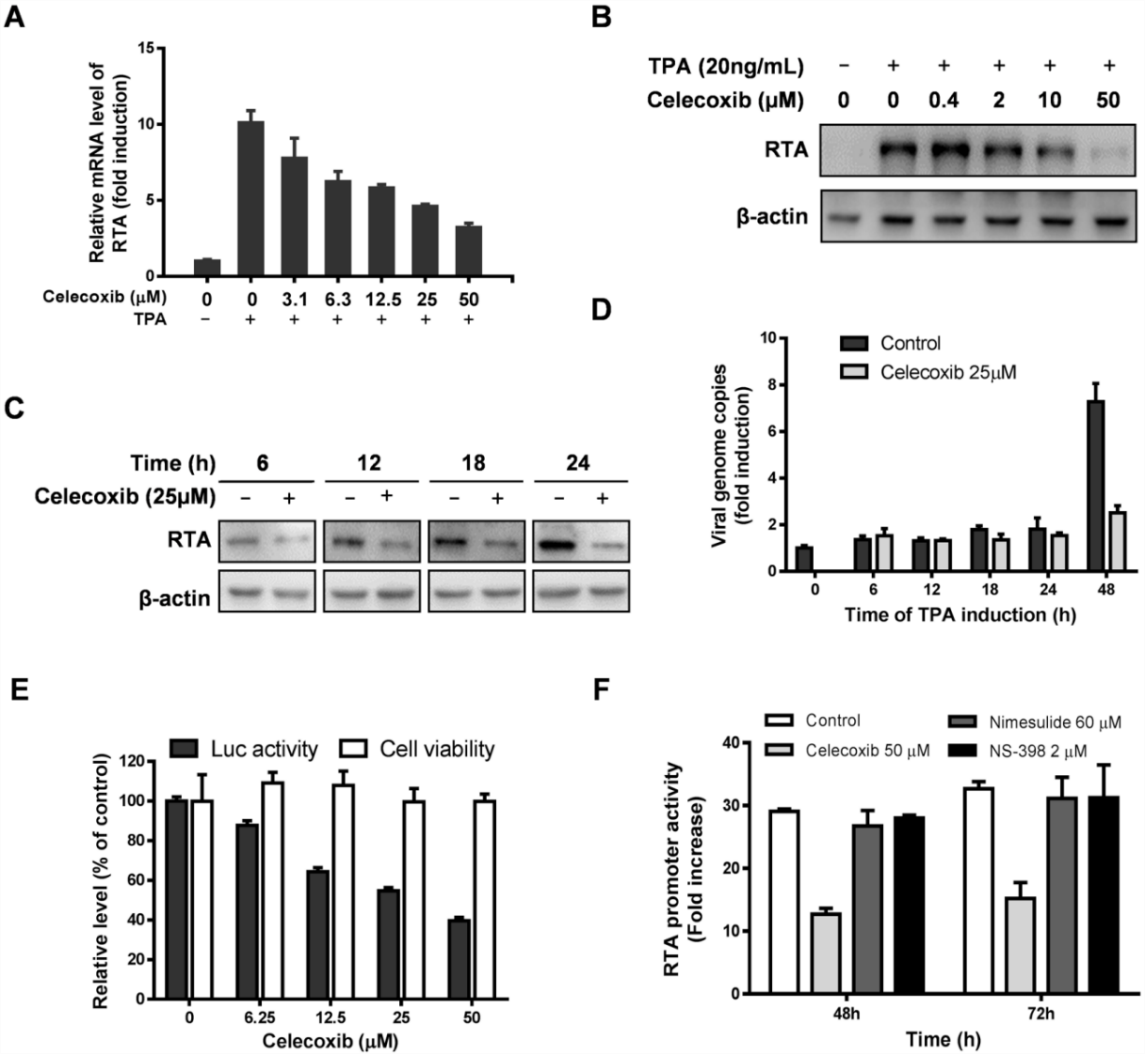
Celecoxib suppresses RTA expression through an off-target effect. The levels of RTA mRNA (**A**) and protein expression (**B**) in BCBL-1 treated with celecoxib were determined by WB and RT-qPCR at 72 h after TPA induction respectively. All transcripts of mRNA were normalized to actin gene; (**C**) The effect of celecoxib (25 µM) on RTA expression in BCBL-1 at various time points post TPA induction were determined by WB assay using specific antibodies; (**D**) Kinetics of KSHV genome replication in the presence or absence of celecoxib following TPA induction. The copy number of KSHV genomes was detected by qPCR. Data were normalized as the fold change compared to the TPA un-induced control; (**E**) HEK293 cells co-transfected with pGL3-RTA, pCR3.1-ORF50 and pRL-TK (25:25:1) were treated with various concentrations of celecoxib and the luciferase activity (black bars) was analyzed at 48 h. In parallel with the luciferase assay, the cytotoxicity of HEK293 (grey bars) was determined by CellTiter-Glo^®^ Luminescent Cell Viability Assay; (**F**) HEK293 co-transfected with pGL3-RTA, pCR3.1-ORF50 and pRL-TK in the presence of test compounds was determined by the luciferase activity at (48 h and 72 h). Data were normalized as the relative luciferase activity compared to the DMSO control. The results are presented as the mean values with standard deviations (*n =* 3).

The above results indicate that celecoxib may target the early stages of KSHV reactivation from latency in iSLK.219 cells. Reactivation of KSHV to the lytic cycle requires the expression of early viral genes, which include the master regulator RTA. Moreover, RTA is sufficient for the commencement of lytic replication and the initiation of the lytic protein cascade to produce abundant mature progeny viruses [16,39]. We investigated the effect of celecoxib on the expression of RTA by RT-qPCR and Western blot analysis. Figure 5A,B show that celecoxib dose-dependently reduces the levels of RTA mRNA and protein expression at 72 h post-induction in BCBL-1 cells. The reduction of RTA expression affects the transcription of KSHV lytic genes, so the expression of several lytic genes of KSHV was detected by RT-qPCR. The results showed that celecoxib treatment inhibited the expression of selected IE, E and late genes (Figure S2). To determine whether the inhibitory effect of celecoxib on RTA expression was due to the decreased number of viral genomes, the levels of RTA expression in BCBL-1 treated with 25 µM celecoxib were determined by WB at various time points for RTA expression and amplification of KSHV genomes. As shown in Figure 5C,D, celecoxib reduced RTA expression at all 4 time points before DNA replication compared to the untreated cells. This indicates that the inhibitory effect of celecoxib on RTA expression is not correlated with the reduction of viral DNA replication. To further determine whether celecoxib directly targets the activation of the RTA promoter, transfection of HEK293 cells was performed with pGL3-RTA in the presence of non-toxic concentrations of compound, and the activity of the RTA promoter was measured using a luciferase assay at 48 h post-incubation. As shown in Figure 5E, celecoxib dose-dependently decreased RTA promoter activity, indicating that its inhibitory effect on RTA expression directly regulated RTA promoter activation. Notably, with the incubation of celecoxib (25 µM) over time, the activity of the RTA promoter decreased compared to the increase of the control. However, the COX-2 inhibitors, ns-398 and nimesulide, had a negligible effect on RTA promoter activity at their maximal non-toxic concentrations (Figure 5F), which is consistent with previous studies [40,41], indicating that celecoxib has a specific effect on the regulation of RTA activation.

### 3.5. Celecoxib Affects Phosphorylation of p38 MAPK during KSHV Lytic Phase

**Figure 6:**
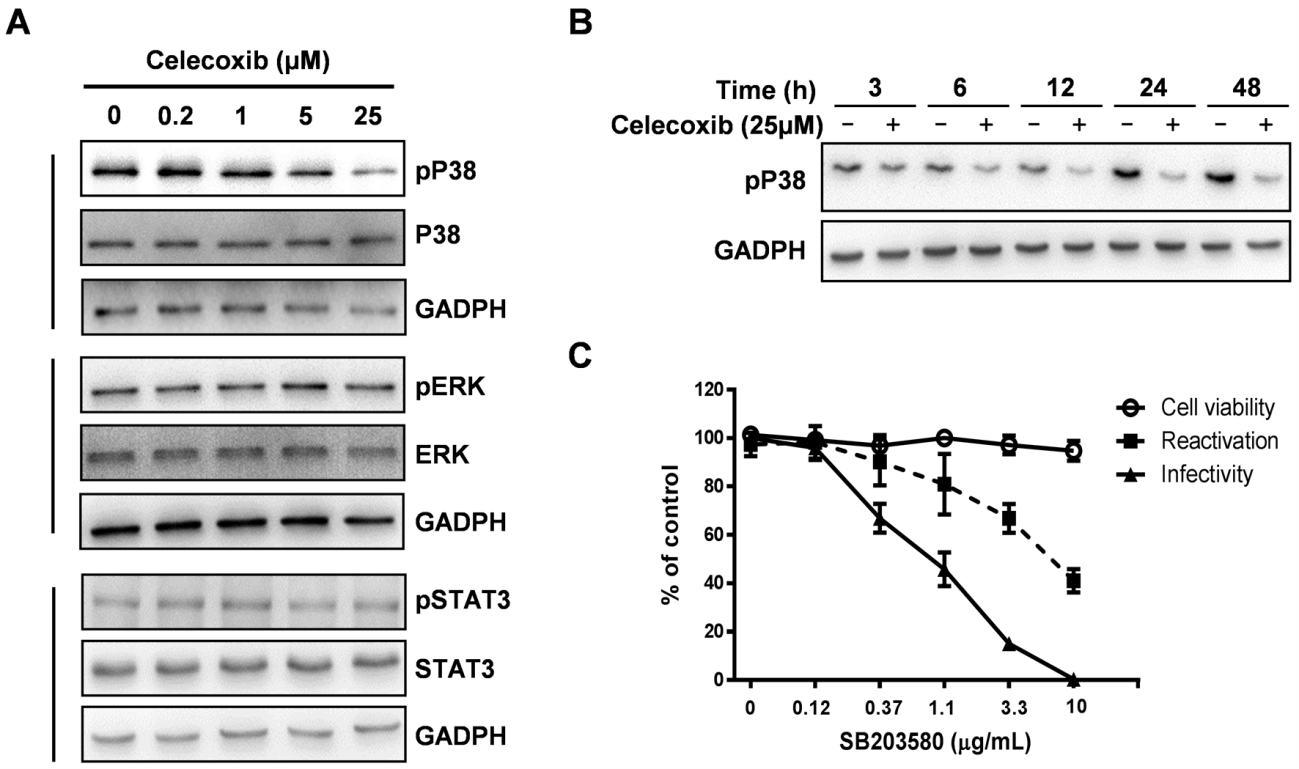
Celecoxib inhibits the activity of the p38 MAPK pathway. (**A**) The effect of celecoxib on test proteins in BCBL-1 was determined at 24 h following TPA induction by WB with specific antibodies; (**B**) Time course analysis of the phosphorylation of p38. BCBL-1 cells treated with celecoxib (25 µM) were collected at the indicated time points after TPA incubation and the phosphorylated form of p38 was detected by WB using a specific antibody. An anti-GADPH antibody was used to normalize the sample loading; (**C**) iSLK.219 cells were treated with SB203580 in the present of Dox and NaB for 48 h. The cytotoxicity of iSLK.219 was measured as described in Methods (○). Then RFP expression (■) in iSLK.219 and the virion infectivity of the supernatant (▲) to 293T was detected and analyzed using the HCS system. Data were normalized as the relative activity compared to the untreated control. The results are presented as mean values with standard deviations (*n =* 3).

The activation of the RTA promoter requires cellular MAPK signaling pathways [16,21]. MAPK pathways, including the MEK/ERK, JNK, and p38 pathways, modulate the activity of the RTA promoter by regulating AP-1 signaling [20]. However, the induction of the KSHV lytic replication by TPA treatment enhances the levels of activated ERK and p38, but not activated JNK [22], suggesting that the regulation of ERK and p38 may be more flexible and related to lytic replication. Previous studies suggested that celecoxib reduced TPA-induced COX-2 expression by blocking the activation of p38 MAPK and AP-1 in female ICR mouse skin [42]. To clarify the roles of p38 and ERK in celecoxib-inhibited KSHV activation, BCBL-1 cells were exposed to celecoxib in the presence of TPA and then harvested at 24 h to assess the levels of ERK and p38 phosphorylation by WB assay. As shown in Figure 6A (upper and middle panels), the phosphorylated form of p38 was dose-dependently decreased by celecoxib compared to the steady-state level of the p38 and GAPDH proteins; however, the phosphorylated form of ERK was not affected. The inhibitory effect of celecoxib on the phosphorylation of p38 was also tested in iSLK.219 cells, celecoxib displayed the similar inhibitory effect compared to that in BCBL-1 cells (Figure S4A). This indicates that celecoxib reduced the levels of phosphorylated p38 MAPK during the KSHV lytic phase. Next, to determine whether inhibition of p38 phosphorylation simply indicates a global shutdown of all protein phosphorylation within the treated cells, we assessed the effects of celecoxib on the signal transducer and activator of transcription 3 (STAT3), which is constitutively phosphorylated in PEL cell lines and plays a role in transcription and cellular functions [43,44]. As shown in Figure 6A (bottom panel), the levels of the phosphorylated form of STAT3 remained unchanged, indicating that celecoxib did not induce global dysregulation of phosphorylation and cytotoxicity for cell growth. To clarify whether the phosphorylation of p38 is dependent on the COX-2 activity, two other COX-2 inhibitors, NS-398 and nimesulide, were tested for activity on the phosphorylation of p38. The results showed that NS-398 and nimesulide did not affected the phosphorylation of p38 MAPK in BCBL-1 (Figure S3) and iSLK.219 cells (Figure S4B,C), suggesting that the inhibitory effect of celecoxib on p38 activation is independent of the COX-2 pathway.

Furthermore, we detected the level of phosphorylated p38 in cells treated with celecoxib at the maximum non-toxic concentration (25 µM) by WB at various time points after TPA-induction (Figure 6B). The result confirmed the inhibitory effect of celecoxib on p38 activation. Additionally, the selective inhibitor of p38 MAPK, SB203580, dose-dependently reduced RFP expression in iSLK.219 cells and decreased the infectivity of the supernatant to 293T cells at non-toxic concentrations compared to the control (Figure 6C), suggesting that the activity of p38 is necessary for KSHV reactivation and the production of infectious virions.

Because the inhibition of activity of p38 MAPK suppresses KSHV activation, we conclude that celecoxib inhibits the lytic replication of KSHV by decreasing the expression of RTA by blocking the activation of p38 MAPK.

## 4. Discussion

In this study, the antiviral activity of celecoxib was examined in two different cells, BCBL-1 and iSLK.219. Despite of the use of different agents for the induction of KSHV lytic replication in BCBL-1 and iSLK.219 cells, the antiviral activity of celecoxib targets lytic replication of KSHV regardless of the inducers and cell types. The copy number of KSHV episomes and the levels of LANA expression were not impaired by celecoxib, indicating that the decrease of viral DNA copies in lytic replication by celecoxib is not the cause of loss of latent KSHV episomes in cells. Although antiviral data indicate that the SI values are relatively medium, the antiviral activity of celecoxib was confirmed in a variety of assays and cannot be attributed to unspecific cell pathogenic effects.

To elucidate the antiviral mechanism of celecoxib, we used iSLK.219 cells to determine the stages of the KSHV life cycle that celecoxib targets. The cells contain RFP as a marker of viral lytic early gene expression and produce abundant infectious virions [26,27]. The expression level of RFP, the viral DNA copy number and the production of infectious virions artificially divide the viral lytic cycle into three phases, the early stages of reactivation, DNA replication and virion production. The addition of celecoxib effectively decreased RFP expression, which is similar to the inhibitory profile of SP600125, but not CDV, which did not inhibit RFP expression (Figure 3). In addition, the earlier celecoxib were added, the higher inhibitory effects were observed (Figure S1), further indicating that celecoxib inhibits the early stage(s) of KSHV reactivation. Because RTA is an important regulator of the early stages of KSHV reactivation, we investigated the effect of celecoxib on RTA expression. The results from RT-qPCR, WB and luciferase reporter assays showed that celecoxib specifically reduced RTA expression (Figure 5), explaining its mechanism of action for the lytic reactivation of KSHV.

The activity of the RTA promoter depends on the activation of a number of specific cellular signaling pathways. Our data show that the mechanism for the inhibitory effect of celecoxib on RTA expression is by blocking p38 activation. The negligible effect of celecoxib on the expression and phosphorylation of ERK and STAT3 indicates that the suppression of p38 MAPK is not a result of global cellular dysregulation (Figure 6). Reactivation of KSHV from latency is modulated by the MAPK pathway, which includes ERK, JNK and p38, and inhibitors of the MAPK pathways prevent KSHV activation. The MAPK pathways regulate KSHV reactivation through AP-1, which binds to specific sites of the RTA promoter to initiate expression and activate the KSHV lytic replication program [20,21,22,24]. Notably, celecoxib, which inhibits activation of AP-1 and p38 MAP kinase in mouse skin [42], further strengthens our prediction that celecoxib reduces RTA expression, thereby leading to inhibition of the lytic gene cascade by inhibiting p38 phosphorylation. In addition, celecoxib does not inhibit KSHV latent replication suggests that the p38 phosphorylation may not be involved in KSHV latent replication. Although the precise molecular mechanism of celecoxib against KSHV lytic replication remains to be elucidated, including targets of celecoxib, such as cyclooxygenase-2 (COX-2), Ca^2+^ATPase, protein dependent kinase 1 (PDK-1), TNF-α, NF-κB, and STAT3 [45,46,47], our results demonstrate that celecoxib reduces the activity of p38 required for KSHV lytic replication and lytic transcription.

The majority of recent studies showed that celecoxib has a potent anticancer function against various human neoplasms and inhibits virus infection in a COX-2 dependent or independent manner [45,48,49]. However, COX-2 participates in the regulation of virus replication and the modulation of inflammatory responses following infection with HSV-1, EBV, KSHV and CMV [50,51,52]. Previously, studies found that during KSHV *de novo* infection of endothelial cells, COX-2 is a highly up-regulated gene activated by the oncoprotein-v-FLIP, and it plays multiple roles in the establishment and maintenance of KSHV latency. Treatment with the COX-2 inhibitors can suppress KSHV primary infection, establishment of latency and the proliferation of KSHV-infected cells [40,41,52,53,54]. However the effects of COX-2 and its inhibitors on KSHV lytic replication are unclear. We employed the TPA-induced BCBL-1 cells to test the antiviral activity of celecoxib against KSHV lytic replication. At the non-toxic concentrations, celecoxib inhibits KSHV lytic replication. A negative effect of celecoxib on viral DNA load and LANA expression in latency indicated that the antiviral activity of celecoxib is specific against KSHV lytic replication (Figure 1). Notably, the expression and activity of ERK and STAT3 remained unchanged, indicating that the inhibitory effect of celecoxib is not due to global dysregulation (Figure 6A). Interestingly, other inhibitors of COX-2, NS-398 and nimesulide, have minimal effect on KSHV DNA replication (Figure 2), reactivation (Figure 4) and the activity of the RTA promoter (Figure 5F), suggesting that the lytic replication of KSHV may be independent of the COX-2 pathway and that the inhibitory effect of celecoxib on the lytic replication of KSHV is via an off-target effect.

Recently, the treatments for KSHV-associated malignancies remain toxic and incompletely efficacious. Therefore, the discovery and development of novel therapeutics for KSHV infection is warranted. In this study, we found that celecoxib inhibits lytic replication through regulating RTA expression by blocking the activation of MAPK p38. Celecoxib, the first FDA approved COX-2-selective inhibitor, initially developed for the clinical treatment of rheumatoid arthritis and relief of inflammation and pain, has also been used for the treatment of familial adenomatous polyposis [55,56,57,58]. However, severe cardiovascular and gastrointestina complications limited clinical application of the coxib class of inhibitors such as rofecoxib and valdecoxib, but celecoxib is widely used in patients with osteoarthritis and rheumatoid arthritis because it causes less side effects than traditional NSAIDs [59,60]. Celecoxib has been extensively used in the clinic, it may serve as an alternative therapeutic for KSHV-related malignancies because of its relative drug safety.

## 5. Conclusions

In this study, we demonstrate that celecoxib significantly inhibits KSHV reactivation, and the inhibitory effect is mediated via a COX-2-independent pathway. Celecoxib targets at early stages of KSHV reactivation by reducing the expression of immediate-early gene RTA through blocking the phosphorylation of MAPK p38.

## Supplementary Files

Supplementary File 1
