# Supplementary material for: Celecoxib Inhibits the Lytic Activation of Kaposi’s Sarcoma-Associated Herpesvirus through Down-Regulation of RTA Expression by Inhibiting the Activation of p38 MAPK
Source: Viruses. 2015 May 5;7(5):2268–87. doi: 10.3390/v7052268 (PMC4452905; doi:10.3390/v7052268)
Supplement: Supplementary File 1 [file viruses-07-02268-s001.pdf]

# Supplementary Figures

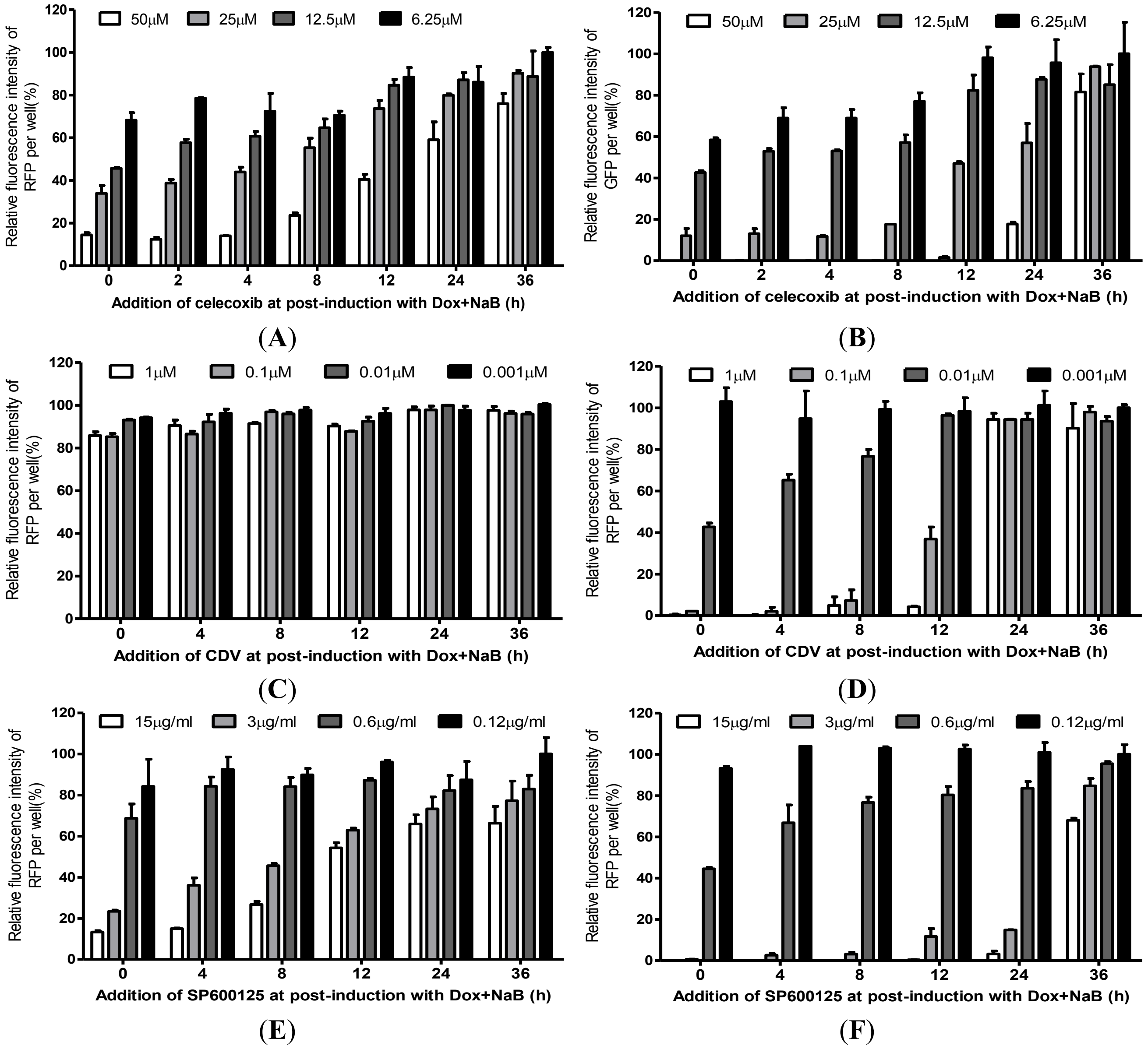


**Figure S1.** Time-of-drug addition approach to define the antiviral mechanism of celecoxib. The induced iSLK.219 cells were treated with test compounds at onset of Dox and NaB induction or at 4, 8, 12, 24 and 36 h post-induction. And at 48 h post-induction, the effects of celecoxib (**A**); CDV (**C**) or SP600125 (**E**) on RFP expression in iSLK.219 cells were detected and analyzed by HCS. Then the supernatants were collected to infect the 293T cells as the method section described. At 48h post-infection, the effects of celecoxib (**B**); CDV (**D**) or SP600125 (**F**) on GFP expression were detected and analyzed by the Operetta HCS system. Images from nine fields per well were digitized to determine the total intensity using the Harnomy3.5 software. Data were normalized as the relative fluorescence intensity compared to the DMSO control. The results are presented as the mean values with standard deviations (*n* = 3).

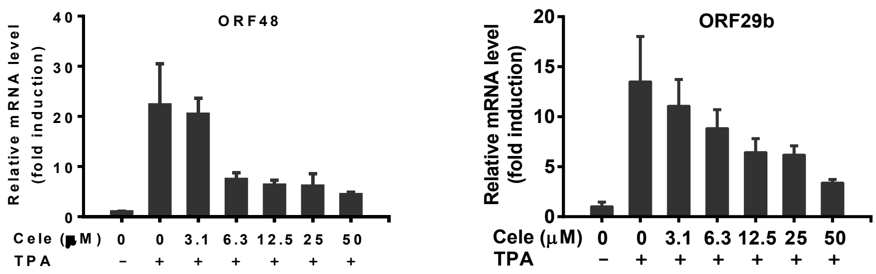


(A)

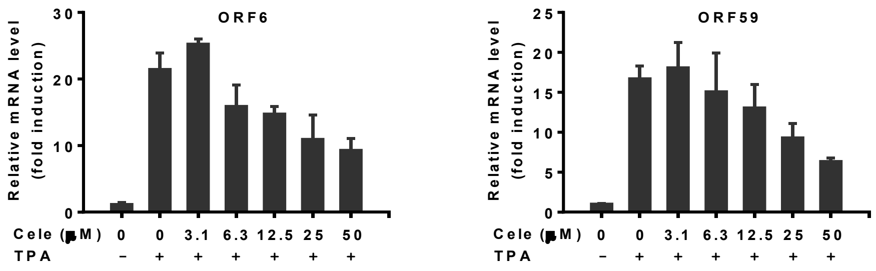


(B)

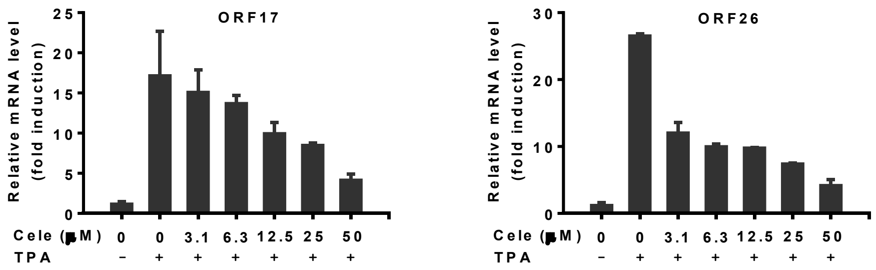


(C)

**Figure S2.** Celecoxib treatment decreases the expression of selected lytic genes. BCBL-1 cells were treated with various concentrations of celecoxib for 72 h following TPA induction. Then, RT-qPCR were performed to determine the levels of mRNA expression of immediate-early genes orf48 and orf29b (**A**); early genes orf6 and orf59 (**B**); and late genes orf17 and orf26 (**C**). Actin was used as the loading controls. Data were normalized as the fold induction compared to the no-TPA inducted control. The results are presented as the mean values with standard deviations ($n$ = 3).

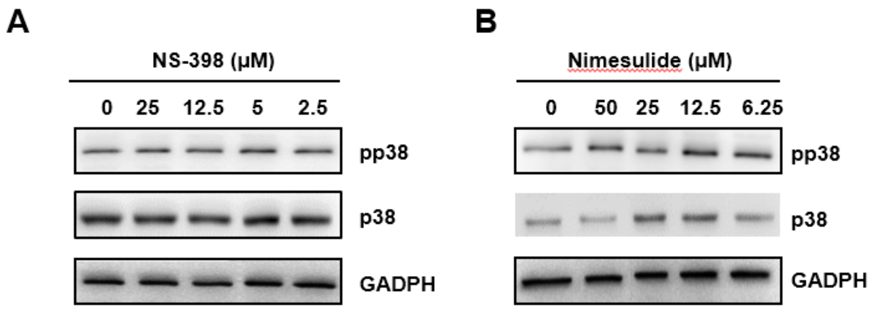


**Figure S3.** COX-2 inhibitors, NS-398 and nimesulide, have no effect on phosphorylation of p38 MAPK in BCBL-1 cells. The effects of NS-398 (**A**) and nimesulide (**B**) on the expression of p38 and pp38 in BCBL-1 were determined at 24 h following TPA induction by WB with specific antibodies. An anti-GADPH antibody was used to normalize the sample loading.

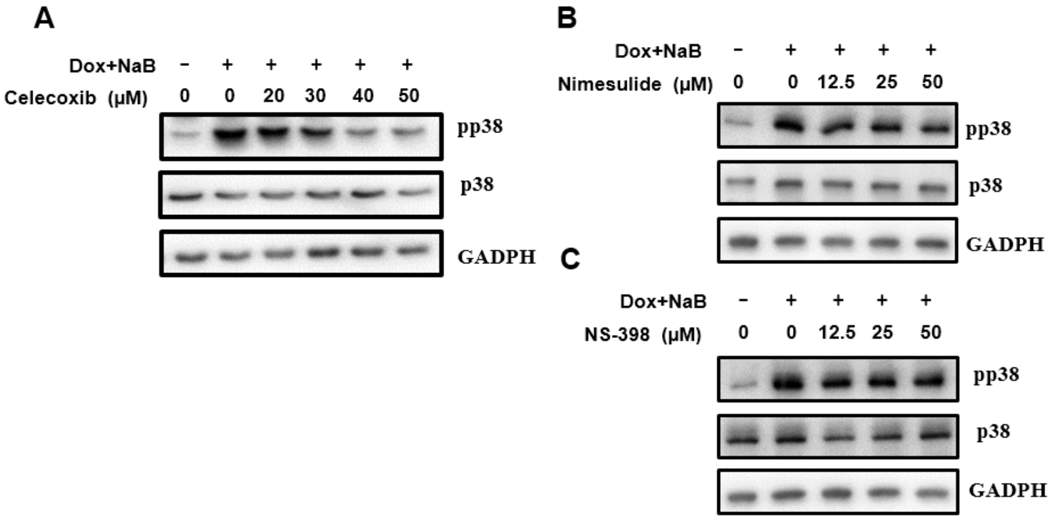


**Figure S4.** Celecoxib but not NS-398 and Nimesulide affects phosphorylation of p38 MAPK in iSLK.219 cells. The effects of celecoxib (**A**); NS-398 (**B**) and (**C**) on the expression of p38 and pp38 in iSLK.219 cells were determined at 24 h following Dox and NaB induction by WB with specific antibodies. An anti-GADPH antibody was used to normalize the sample loading.
